# Enzyme catalysis captured using multiple structures from one crystal at varying temperatures

**DOI:** 10.1107/S205225251800386X

**Published:** 2018-03-16

**Authors:** Sam Horrell, Demet Kekilli, Kakali Sen, Robin L. Owen, Florian S. N. Dworkowski, Svetlana V. Antonyuk, Thomas W. Keal, Chin W. Yong, Robert R. Eady, S. Samar Hasnain, Richard W. Strange, Michael A. Hough

**Affiliations:** a School of Biological Sciences, University of Essex, Wivenhoe Park, Colchester CO4 3SQ, England; b Scientific Computing Department, STFC Daresbury Laboratory, Warrington WA4 4AD, England; c Diamond Light Source, Harwell Science and Innovation Campus, Didcot OX11 0DE, England; d Swiss Light Source, Paul Scherrer Institute, 5232 Villigen PSI, Switzerland; e Molecular Biophysics Group, Institute of Integrative Biology, University of Liverpool, Crown Street, Liverpool L69 7ZB, England

**Keywords:** serial crystallography, copper nitrite reductase, variable temperature, radiolysis, structural dynamics, density functional theory

## Abstract

High-resolution crystal structures of enzymes in relevant redox states have transformed our understanding of enzyme catalysis. Recent developments have demonstrated that X-rays can be used, *via* the generation of solvated electrons, to drive reactions in crystals at cryogenic temperatures (100 K) to generate ‘structural movies’ of enzyme reactions. However, a serious limitation at these temperatures is that protein conformational motion can be significantly supressed. Here, the recently developed MSOX (multiple serial structures from one crystal) approach has been applied to nitrite-bound copper nitrite reductase at room temperature and at 190 K, close to the glass transition. During both series of multiple structures, nitrite was initially observed in a ‘top-hat’ geometry, which was rapidly transformed to a ‘side-on’ configuration before conversion to side-on NO, followed by dissociation of NO and substitution by water to reform the resting state. Density functional theory calculations indicate that the top-hat orientation corresponds to the oxidized type 2 copper site, while the side-on orientation is consistent with the reduced state. It is demonstrated that substrate-to-product conversion within the crystal occurs at a lower radiation dose at 190 K, allowing more of the enzyme catalytic cycle to be captured at high resolution than in the previous 100 K experiment. At room temperature the reaction was very rapid, but it remained possible to generate and characterize several structural states. These experiments open up the possibility of obtaining MSOX structural movies at multiple temperatures (MSOX-VT), providing an unparallelled level of structural information during catalysis for redox enzymes.

## Abstract 2

MSOX (multiple serial structures from one crystal) serial crystallography experiments were carried out using controlled X-ray radiolysis and photon-counting detectors to determine sequences of low-dose yet high-resolution structures of copper nitrite reductase. Working at 190 K and at room temperature provides greater dynamic freedom, allowing more of the catalytic cycle to be observed than at the usual cryogenic temperature of 100 K. The approach demonstrates the potential to obtain MSOX structural movies at variable temperatures, thus providing an unparalleled level of structural information during catalysis for redox enzymes.

## 1. Introduction   

High-resolution protein structures determined by X-ray crystallography are central to our understanding of many biological processes. Almost all such structures are currently determined at a temperature of 100 K in order to limit the effects of X-ray radiation damage and extend crystal lifetimes by a factor of ∼100. It is increasingly becoming recognized that protein functional dynamics may be suppressed or altered by cryocooling and that protein crystals undergo dynamic transitions at cryogenic temperatures above 100 K (Weik & Colletier, 2010 ▸; Weik *et al.*, 2001 ▸). The development of fast photon-counting detectors allowing shutterless data collection has recently led to a ‘room-temperature renaissance’ where single data sets may be measured rapidly at ambient temperature prior to crystal destruction (Owen *et al.*, 2012 ▸, 2014 ▸). Crystallography performed at room temperature and elevated cryogenic temperatures may reveal a greater range of conformational freedom for enzymes to undergo catalysis or ligand binding in crystals (Fraser *et al.*, 2009 ▸, 2011 ▸; Fischer *et al.*, 2015 ▸).

We previously demonstrated that solvated electrons produced by X-ray irradiation (Berglund *et al.*, 2002 ▸) can be used to initiate conversion of the nitrite substrate to the NO product in cryocooled (100 K) *Achromobacter cycloclastes* copper nitrite reductase (*Ac*NiR) crystals, producing a ‘structural movie’ from 45 consecutive structures measured from the same crystal volume (Horrell *et al.*, 2016 ▸). The reduction of nitrite to NO is a critical step in the microbial denitrification pathway since it is at this point that terrestrial fixed nitrogen is lost to the atmosphere, with consequent significance for the environment and agriculture.

Copper nitrite reductases (CuNiRs) are typically trimeric, with each monomer comprising two cupredoxin domains, a type 1 copper (T1Cu) site with an electron-transfer role and a catalytic type 2 copper (T2Cu) site, which are separated by ∼12.6 Å *via* a Cys–His bridge (Horrell *et al.*, 2017 ▸). Extensive spectroscopic and structural studies of CuNiRs (Strange *et al.*, 1999 ▸; Boulanger *et al.*, 2000 ▸; Boulanger & Murphy, 2001 ▸; Tocheva *et al.*, 2004 ▸, 2007 ▸; Antonyuk *et al.*, 2005 ▸; Krzemiński *et al.*, 2011 ▸; Wijma *et al.*, 2006 ▸; Hough *et al.*, 2008 ▸; Leferink *et al.*, 2011 ▸, 2012 ▸) have suggested a general catalytic mechanism in which bound water in the resting state is replaced by nitrite, and internal electron transfer occurs between the T1Cu and T2Cu sites. Two protons are required for the reaction, which are thought to be provided by Asp98 (Asp_CAT_) and His255 (His_CAT_) *via* a bridging water molecule, leading to cleavage of the bound NO_2_^−^ to produce NO and H_2_O. Conformational rearrangements of Asp_CAT_ have been proposed to be important in substrate guidance and proton transfer (Antonyuk *et al.*, 2005 ▸), while His_CAT_ may respond to the redox state. Notably, the NO binding geometry observed in crystal structures is side-on, with near-equidistant Cu—N and Cu—O bonds (Antonyuk *et al.*, 2005 ▸; Tocheva *et al.*, 2004 ▸), which is in conflict with computational and spectroscopic studies, which predict an end-on mode (Maekawa *et al.*, 2015 ▸), and it remains an open question as to what extent the binding geometry is affected by temperature and differences in dynamics. The resting-state water-bound T2Cu is restored following release of NO from the active site.

Here, we describe MSOX (multiple serial structures from one crystal) structural movies obtained at 190 K and room temperature (RT) to explore the effect of enzyme dynamic freedom on catalysis in crystals. In contrast to the previous movie at 100 K, these data reveal many more structural processes/steps relevant to catalysis: reorientation of nitrite, conversion of nitrite to NO, release of NO and rebinding of water to the catalytic T2Cu atom, followed by final reduction to a T2Cu(His)_3_ state. This variable-temperature MSOX approach is widely applicable for obtaining structural movies capturing redox-enzyme reactions at high resolution from a single crystal. This adds to the serial crystallography approaches that have been pioneered at X-ray free-electron lasers (Spence & Lattman, 2016 ▸; Levantino *et al.*, 2015 ▸; Chapman *et al.*, 2011 ▸).

## 2. Materials and methods   

### 2.1. Sample preparation and measurement of MSOX data   

Recombinant *Ac*NiR protein and crystals were both prepared as described by Horrell *et al.* (2016 ▸) and Antonyuk *et al.* (2005 ▸), respectively. Crystals (approximate dimensions 300 × 200 × 200 µm) were mounted in loops for data collection at 190 K and in a MiTeGen RT capillary system for room-temperature data collection. The 190 K MSOX data were measured on SLS beamline X10SA with an X-ray wavelength of 0.99 Å and a 100 × 100 µm beam using a PILATUS 6M-F detector. The crystals were initially cooled to 100 K by plunging them into liquid nitrogen for transport and were mounted on the beamline in a cryostream at 100 K before the cryojet temperature was raised to 190 K for data collection. Optical data were measured *in situ* before and after each of the first 15 X-ray data sets using the on-axis MS3 single-crystal microspectrophotometer. Each complete data set of 300 frames, each of 0.1 s exposure, was recorded in 30 s, with the whole series, comprising 22 500 images in 75 data sets, taking approximately 90 min, including dead time. The X-ray flux was 1.05 × 10^11^ photons s^−1^ for the first 15 data sets and was gradually increased to 9.30 × 10^11^ photons s^−1^ to drive the reaction to completion. Data were processed using *XDS* (Kabsch, 2010 ▸) and *AIMLESS* (Evans & Murshudov, 2013 ▸). Room-temperature data were measured on beamline I24 at Diamond Light Source with a PILATUS3 6M detector, using an X-ray wavelength of 0.8 Å with a defocused 30 × 30 µm beam and an X-ray flux of 1.6 × 10^11^ photons s^−1^. Each data set of 300 frames with 0.01 s exposure per frame (100 Hz detector readout) was collected in 3 s total exposure time, with the whole series comprising ten data sets and 3000 frames measured over 30 s total exposure. A 14 s interval between successive data sets in this series was unavoidable^1^ with the current software setup on beamline I24, and this resulted in an extension of the total data-collection time to ∼3 min. Data were processed using *DIALS* (Winter *et al.*, 2018 ▸) within *xia*2 (Winter *et al.*, 2013 ▸). Diffraction data were recorded from the same illuminated crystal volume in each series. The series were processed with high-resolution cutoff criteria of CC_1/2_ > 0.3 (190 K), CC_1/2_ > 0.5 (RT) (Karplus & Diederichs, 2012 ▸) and *I*/σ(*I*) > 1.0 for the highest resolution shell. All structures were refined based on a starting model of the 100 K *Ac*NiR–nitrite complex at 1.07 Å resolution with the ligand removed (PDB entry 5i6k; Horrell *et al.*, 2016 ▸). Refinement was performed by maximum-likelihood methods in *REFMAC*5 (Murshudov *et al.*, 2011 ▸) within the *CCP*4 suite v.7 (Winn *et al.*, 2011 ▸) using the *CCP*4*i*2 interface (Potterton *et al.*, 2018 ▸). Anisotropic temperature factors were used to model the 190 K data sets with resolutions of ≤1.4 Å and isotropic temperature factors were used during refinement of all room-temperature data. Cu–ligand distances and bond angles were unrestrained during refinement. *Coot* (Emsley *et al.*, 2010 ▸) was used for model building using the 2*F*_obs_ − *F*_calc_ and *F*_obs_ − *F*_calc_ electron-density maps. The occupancies of ligands, amino-acid side chains and solvent at the active site were assessed using *F*_obs_ − *F*_calc_ OMIT maps and the consistency of their *B* factors. The free *R* factor (Brünger, 1992 ▸) was calculated from 5% of data excluded from the refinement. Average absorbed X-ray doses were estimated using the diffraction-weighted dose metric of *RADDOSE*-3*D* (Zeldin *et al.*, 2013 ▸) with a Gaussian beam profile. The data-reduction and refinement parameters are given in Supplementary Tables S1 and S2. Figures and movies were generated in *PyMOL* (v.1.8; Schrödinger) or *Molden* (Schaftenaar & Noordik, 2000 ▸).

### 2.2. DFT modelling of oxidized and reduced T2Cu–nitrite complexes   

Density functional theory (DFT) calculations employed crystal structures from the movie frames showing the top-hat and side-on modes of binding of nitrite at the T2Cu site. The starting models consisted of the T2Cu atom, the nitrite ligand, the three histidine ligands (His100, His135 and His306), Asp98 (in its proximal position) and His255, both of which are implicated in the proton-transfer pathway (Kataoka *et al.*, 2000 ▸; Boulanger *et al.*, 2000 ▸), the hydrophobic residue Ile257 that plays a potential role in shaping the substrate-binding pocket (Boulanger & Murphy, 2003 ▸; Tocheva *et al.*, 2008 ▸), and two crystallographic water molecules. One water molecule forms a hydrogen-bond bridge between Asp98 and His255, and the other forms part of the conserved water channel and is the closest water to nitrite in the crystal structure. Both Asp98 and His255 were protonated in accordance with the crystallization conditions (pH 5) and consistent with the protonated states used by Ghosh and coworkers at low pH (Ghosh *et al.*, 2009 ▸). The serial structures revealed little variation in the positions of the active-site residues. The model structures were therefore constrained accordingly to preserve the metal-binding environments close to the crystal structures. The models were prepared in two steps. Firstly, H atoms were added to the crystal structure to satisfy the valency and optimized, keeping the heavy atoms fixed. In the subsequent step, the nitrite, the two water molecules and the two protons added to protonate Asp98 and His255 were optimized. This was carried out for both the copper(I) and copper(II) oxidation states. The B3LYP functional was used for optimization together with the DFT-D3 dispersion correction (Grimme *et al.*, 2010 ▸). The def2-TZVP basis set was used for Cu atoms and the def2-SVP basis set was used for other atoms (Weigend & Ahlrichs, 2005 ▸). The optimizations were carried out using the DL-FIND geometry-optimization library (Kästner *et al.*, 2009 ▸) in *ChemShell* (Sherwood *et al.*, 2003 ▸; Metz *et al.*, 2014 ▸) interfaced to the *ORCA* package (Neese, 2012 ▸) for DFT calculations. Structures were generated for alternate protonation states of Asp98 and the alternative positions of nitrite in top-hat coordination, and the lowest energy structures were selected for further analysis. All of the optimized structures and their absolute energies are given in the Supporting Information. The nudged elastic band method (Henkelman *et al.*, 2000 ▸) implemented in DL-FIND was used to calculate the minimum energy path between the optimized top-hat nitrite–T2Cu(I) and side-on nitrite–T2Cu(I) structures. To ensure a consistent energy surface, the nitrite ligand, two water molecules and the H atoms of Asp98 and His255 were optimized while all other atoms were fixed, and the same functional and basis sets were used as for the endpoint minimizations.

## 3. Results   

### 3.1. MSOX series at 190 K   

**Figure 1:**
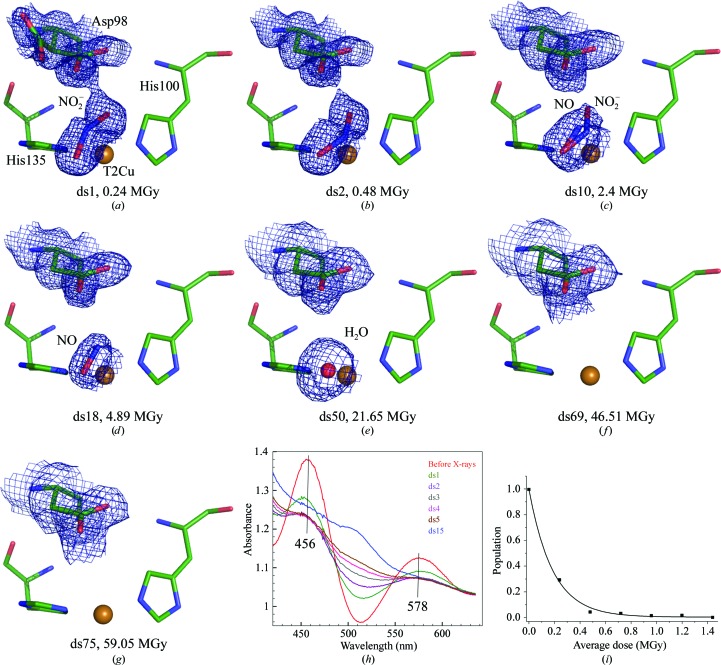
MSOX movie determined at 190 K. The 75-frame structural movie captures multiple states of the enzyme catalytic cycle: (*a*, *b* and *c*) conversion of nitrite to nitric oxide, (*d*) the fully nitric oxide-bound state, (*e*) reformation of the water-bound resting state, and (*f* and *g*) final reduction to the copper(I)–His_3_ state. (*a*) Nitrite is bound in the ‘top-hat’ orientation at ds1_190_ with a double conformation of Asp98 in the proximal and gatekeeper positions. (*b*) The ‘top-hat’ nitrite immediately switches to the ‘side-on’ orientation in ds2_190_ (Asp98 is present only in the proximal orientation) and nitrite only is observed until ds9_190_. (*c*) Nitric oxide is observed at ds10_190_ together with nitrite, (*d*) nitric oxide is fully present by ds18_190_ and is replaced by water at ds50_190_ and (*e*) water remains bound until ds69_190_. (*f*) and (*g*) Data sets 69–75 have no ligand bound to the T2Cu site. The 2*F*_obs_ − *F*_calc_ density in e Å^−3^ is contoured at 0.57 (ds1_190_), 0.55 (ds2_190_), 0.46 (ds10_190_), 0.42 (ds18_190_), 0.40 (ds50_190_), 0.43 (ds69_190_) and 0.42 (ds75_190_). The cumulative X-ray dose absorbed by the crystal is indicated for each data set of the MSOX series. (*h*) Single-crystal UV–Vis measurements before any X-ray exposure and between X-ray data collections until ds15_190_. The first spectrum (red) confirmed the oxidized state of the protein crystal prior to ds1_190_, with absorption maxima at 456 and 578 nm. Significant reduction of the T1Cu site was observed to occur after completion of the ds1_190_ data collection (green spectrum), with the site predominantly reduced by ds2_190_. Full reduction was observed well before ds15_190_ (blue spectrum). Note that a progressive background shift and higher absorbance at low wavelengths is observed with continued irradiation, consistent with previous studies. (i) Dose-dependence of T1Cu reduction, using the change in absorbance at 578 nm to show the fractional change in population.

Using a single nitrite-bound *Ac*NiR crystal at 190 K, an MSOX series of 75 serial structures was obtained (Supplementary Table S1), during which the high-resolution limit decreased from 1.08 Å in the first data set (ds1_190_) to 1.84 Å in ds75_190_. The initial structure (ds1_190_) was superimposable with the previous 100 K MSOX starting structure, ds1_100_ (Horrell *et al.*, 2016 ▸), with an all-protein-atom r.m.s.d. of 0.1 Å. Nitrite was observed bound at T2Cu in a single ‘top-hat’ (*i.e.* vertical bidentate) conformation (Fig. 1 ▸ *a*), with the O atoms of nitrite coordinated to T2Cu at distances of 2.03 and 1.97 Å. A similar top-hat nitrite-binding geometry was previously observed as one of two nitrite orientations in the first structure of our 100 K *Ac*NiR MSOX series (Horrell *et al.*, 2016 ▸). The crucial active-site residue Asp98 (Asp_CAT_) has two alternate conformations (proximal and gatekeeper) with occupancies of 0.75 and 0.25, respectively, consistent with our earlier 100 K MSOX data. For the 190 K series, single-crystal visible spectra (Figs. 1 ▸ *h* and 1 ▸ *i*) were measured before and after each of the initial 15 data sets to monitor the oxidation state of T1Cu and to correlate this with electron-density changes at the T2Cu site. The spectra show that the T1Cu site is largely reduced following measurement of ds2_190_, with close-to-complete reduction by the end of ds4_190_. T1Cu–ligand distances for both temperature series are summarized in Supplementary Tables S3 and S4.

**Figure 2:**
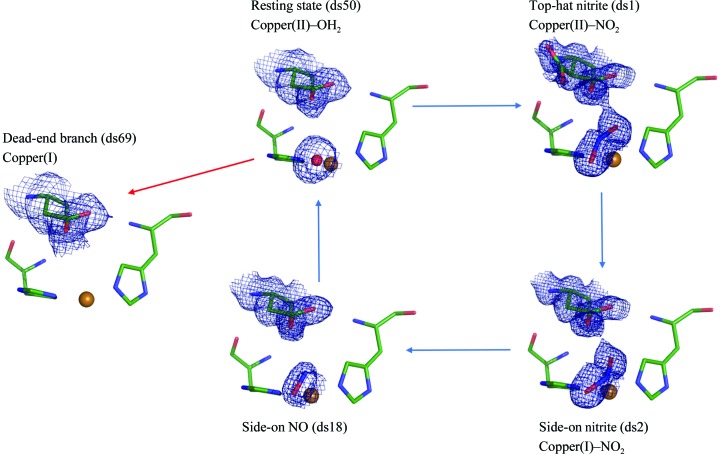
Structures from the proposed nitrite reductase mechanism determined in the 190 K MSOX series. The blue arrows represent the catalytic cycle in which the resting-state enzyme has water bound to T2Cu(II) (ds50). Binding of nitrite and displacement of water forms the initial enzyme–substrate complex T2Cu(II)–NO_2_ (ds1), following which electron transfer from the reduced T1Cu site causes nitrite to switch into its side-on binding mode (ds2). Associated proton transfer produces the side-on NO product (ds18). The red arrow indicates an alternative branch where T2Cu is reduced prior to nitrite binding, resulting in a three-coordinate T2Cu(I) form with (His)_3_ ligation (ds69). This loss of the T2Cu-coordinated water results in loss of enzyme activity.

The nitrite-binding geometry changes from the initial top-hat mode to a side-on orientation (with near-equivalent Cu–O1, Cu–O2 and Cu–N distances) in ds2_190_ (Fig. 1 ▸ *b*). This side-on binding is similar to that present in previous high-dose *Ac*NiR structures (Antonyuk *et al.*, 2005 ▸) and for one of the two nitrite-binding modes observed in the first five structures of the 100 K MSOX series (Horrell *et al.*, 2016 ▸) prior to conversion to NO. From ds2_190_ only the proximal conformation of Asp_CAT_ was visible in the electron-density maps. Notably, the change in nitrite orientation appears at around the same dose as the predominant reduction of the T1Cu site by radiolysis, providing evidence for intramolecular T1Cu–T2Cu electron transfer occurring rapidly within the crystal when the substrate is at the T2Cu site. As further data sets were measured, the side-on T2Cu-bound nitrite was converted into a side-on T2Cu–NO complex following O—N—O bond breakage. By ds18_190_, at 1.32 Å resolution, a full-occupancy NO could be modelled into the electron density (Fig. 1 ▸ *d*). The T2Cu–NO complex persisted in a side-on geometry until ds50_190_, at 1.68 Å resolution, at which point the density was fitted best by a water molecule. Finally, the electron density for the water ligand of T2Cu was absent by ds69_190_, at 1.77 Å resolution, when the catalytic centre was best modelled as a Cu(His)_3_ ligation, consistent with EXAFS and crystallographic data for the fully (chemically) reduced T2Cu(I) form of CuNiRs (Strange *et al.*, 1999 ▸). These structures and their places in the catalytic mechanism are summarized in Fig. 2 ▸. Details of changes to T2Cu coordination and OMIT electron-density maps are given in Supplementary Tables S5 and S6, and Figs. S1 and S2).

### 3.2. Room-temperature MSOX series   

**Figure 3:**
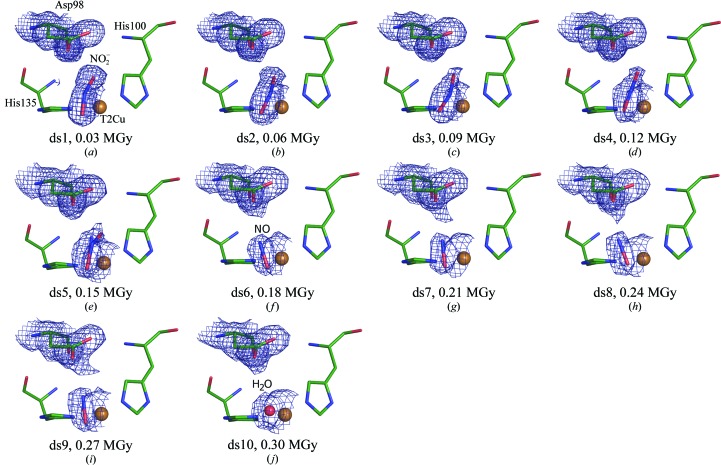
MSOX movie of catalysis in *Ac*NiR at room temperature. A ten-frame X-ray-induced ‘movie’ recorded from consecutive serial data sets showing conversion at the T2Cu site of bound nitrite to NO and then to the H_2_O resting state. Initially in ds1_RT_ the nitrite is bound to T2Cu in the ‘top-hat’ orientation (*a*), but this rapidly changes in ds2_RT_ to a progressively more ‘side-on’ orientation (*b*, *c* and *d*). The ‘side-on’ nitrite is converted to NO by ds5_RT_ (*e*) and the NO is replaced by water at ds10_RT_ (*j*). Only the proximal position of Asp98 is observed in this sequence of electron-density maps. Asp98, His100, His135, nitrite and NO are represented as sticks, and water molecules and Cu atoms as spheres. Other T2Cu-site residues are omitted for simplicity. The 2*F*_obs_ − *F*_calc_ density in e Å^−3^ is contoured at 0.45 (ds1_RT_), 0.43 (ds2_RT_), 0.39 (ds3_RT_–ds7_RT_) and 0.38 (ds8_RT_–ds10_RT_). The cumulative X-­ray dose absorbed by the crystal is indicated for each snapshot of the series.

**Figure 4:**
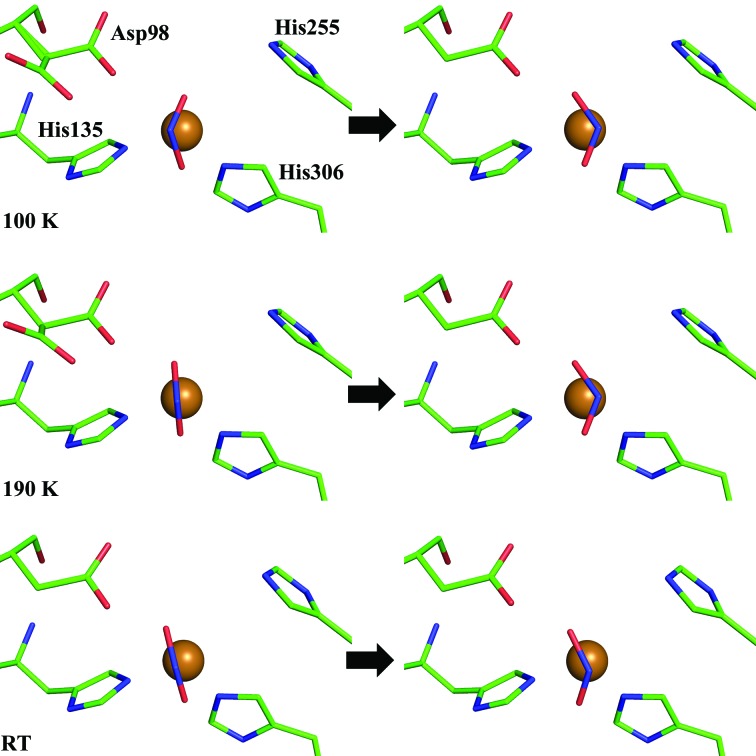
Repositioning of the nitrite ligand in the T2Cu site during catalysis. A switch from the top-hat nitrite (left panels) to the side-on nitrite (right panels) orientation was observed in the first few frames of the MSOX serial movies at 100 K (Horrell *et al.*, 2016 ▸), 190 K and RT. The catalytically important His255 and Asp98 residues are shown, with both the gatekeeper and proximal Asp_CAT_ positions present in the initial structures. The His100 ligand of T2Cu has been omitted for clarity.

A total of ten structures between 1.4 and 1.9 Å resolution were serially obtained from a single *Ac*NiR crystal at room temperature (Supplementary Table S2). In the first structure, ds1_RT_, with an X-ray dose of only 30 kGy, a single top-hat orientation of nitrite was again observed (Fig. 3 ▸ *a*), with oxygen bond lengths to copper of 1.97 and 1.91 Å and a Cu—N bond length of 2.04 Å. The nitrite subsequently re­oriented to adopt a more side-on geometry in ds2_RT_ (Figs. 3 ▸ *b*, 3 ▸ *c* and 3 ▸ *d*) of the series, with a Cu—N bond length of 2.02 Å. Notably, this movement towards the side-on geometry appeared to be less complete in structures at RT (where nitrite rotates by ∼36°) than was observed for the 190 or 100 K MSOX structures (rotation of ∼90°). The repositioning of the nitrite ligand and a comparison of the top-hat and side-on geometries at 100 K, 190 K and RT is given in Fig. 4 ▸. A mixture of nitrite and NO ligands with partial occupancies was modelled in ds5_RT_. The dose-lifetime of this mixture is much shorter than those observed for the MSOX series at 100 and 190 K. At RT, complete conversion of the side-on nitrite to side-on NO was observed by ds6_RT_ (0.18 MGy total dose; Fig. 3 ▸ *f*). NO remained side-on until its release by ds10_RT_, with the enzyme then returning to an apparent resting state with a water molecule bound to T2Cu (Fig. 3 ▸ *j*). A single proximal conformation of Asp_CAT_ was observed throughout this series.

### 3.3. DFT simulations of the T2Cu site   

**Figure 5:**
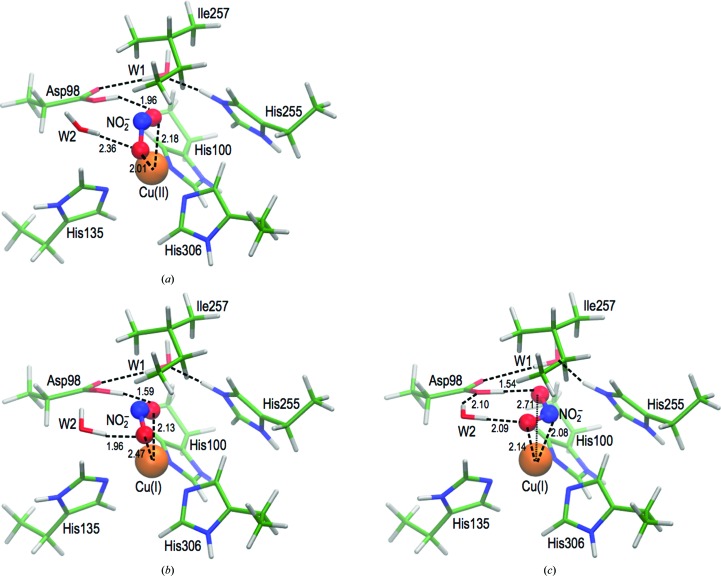
Model DFT structures derived from the crystallographic data for nitrite binding at the T2Cu site. Asp98 (Asp_CAT_) and His255 (His_CAT_) are assumed to be protonated and are linked by hydrogen bonds to the bridging water molecule, W1. W2, which is the closest water to the T2Cu, is part of the highly conserved water network or ‘proton tube’ (not shown here) linking the T1Cu and T2Cu sites to bulk solvent; this is a suggested route for proton delivery during catalysis. The hydrogen-bonding network is indicated by dashed lines, with bond lengths shown in Å. In the T2Cu(II) state (*a*), nitrite was found to relax to the top-hat orientation starting from either the top-hat or side-on crystal structure geometries, indicating that the side-on orientation is not stable in the T2Cu(II) state. In the T2Cu(I) state, the preferred orientation of nitrite after optimization depends on its starting geometry, indicating the presence of a barrier between the top-hat and side-on binding modes. Starting from the top-hat orientation, the relaxed coordination of nitrite at the T2Cu(I) state is an asymmetrical top-hat geometry (*b*); protonation and scission from this orientation are likely to lead to an end-on bound NO at the T2Cu site. Alternatively, starting from the side-on orientation, geometry optimization results in a distorted side-on nitrite coordination (*c*) that is 7.5 kcal mol^−1^ more stable than the asymmetric top-hat orientation in (*b*). The binding geometry in (*c*) is poised for the formation of side-on NO binding, with the nitrite O1 and N atoms bound to the T2Cu at 2.14 and 2.08 Å, respectively, while the O2 atom of nitrite is 2.71 Å from the T2Cu and oriented towards the Asp_CAT_ side chain.

The geometries of the lowest energy optimized structures for the top-hat nitrite–T2Cu(II) state and top-hat and side-on nitrite–T2Cu(I) states obtained from DFT calculations are shown in Fig. 5 ▸. In the oxidized or copper(II) state, nitrite was found to relax to the top-hat orientation starting from either the top-hat or side-on crystal geometries (Fig. 5 ▸ *a*), indicating that the ‘side-on’ orientation is not stable in the copper(II) state. In its reduced or copper(I) state, the preferred orientation of nitrite after optimization depends on its starting geometry, indicating the presence of a barrier between top-hat and side-on binding modes. The minimum energy path (energy barrier) between the two orientations of nitrite for T2Cu(I) was estimated at 1.9–2.4 kcal mol^−1^ using the nudged elastic band method (Supplementary Fig. S3), indicating that the barrier to transition is low. Geometry optimization of the reduced T2Cu site performed using the side-on structures shows that the nitrite ligand shifts from its nearly equidistant side-on Cu—O1/N/O2 ligation to an asymmetric side-on geometry (Fig. 5 ▸ *c*).

## 4. Discussion   

### 4.1. Variable-temperature MSOX data reveal multiple steps of the catalytic cycle   

Within the MSOX series at 190 K and RT, a series of high-resolution, mechanistically relevant structures were determined (Fig. 2 ▸). In both series, nitrite moves between the two binding geometries and is then converted to NO, which proceeds to dissociate from T2Cu and is replaced by a water molecule. A comparison of the T2Cu–water structures determined by MSOX at 100 K, 190 K and RT with the previously determined resting-state structure (PDB entry 2bw4; Antonyuk *et al.*, 2005 ▸) is given in Supplementary Fig. S4. In the previous 100 K MSOX data (Horrell *et al.*, 2016 ▸), both top-hat and side-on nitrite geometries were initially present in the same crystal. A recent 1.6 Å resolution serial femtosecond XFEL (SFX) structure of *Alcaligenes faecalis* CuNiR (*Af*NiR) at room temperature revealed only a top-hat nitrite-binding geometry, while a corresponding RT structure determined using synchrotron radiation (SR) showed a side-on binding mode with nitrite having undergone an ∼55° rotation (Fukuda *et al.*, 2016 ▸; Supplementary Fig. S5). Our 190 K and RT MSOX data revealed that both binding geometries of the ligand are observed in our SR structures, switching between them as dose was accumulated within the crystal. The top-hat geometry observed in the SFX structure and our initial low-dose SR structure represents the initial binding. We suggest that the reorientation of nitrite from top-hat to side-on geometry (Fig. 4 ▸) occurs following electron transfer from the reduced T1Cu and reduction of the T2Cu site. We further propose that the side-on nitrite orientation captured in these crystal structures represents the first step in a repositioning of the bound substrate following electron transfer, prior to proton transfer and cleavage of the nitrite N—O bond and formation of the side-on NO product.

Further understanding is obtained from the DFT simulations of the T2Cu-site environment. The calculations suggest that T2Cu(II) prefers top-hat binding of nitrite, while the side-on conformation observed in the structures corresponds to a T2Cu(I) state. The barrier to transition from top-hat to side-on in the T2Cu(I) state is low and hence the reorientation of nitrite is likely to occur when T2Cu is reduced. The asymmetric side-on geometry found on optimizing the side-on T2Cu structure is appropriate for the subsequent bond cleavage and formation of the side-on NO geometry observed in the later experimental structures (ds18_190_ or ds6_RT_).

Our data therefore suggest that when nitrite is bound, electron transfer from T1Cu results in a T2Cu(I)–side-on nitrite complex poised for conversion to side-on NO. The single-crystal optical data (Fig. 1 ▸) show that by the end of the measurement of ds2_190_ the T1Cu sites bathed by the X-ray beam are essentially fully reduced, and this is concomitant with the appearance of side-on nitrite binding to T2Cu in ds2_190_. The conversion of this side-on nitrite to the side-on NO product appears to occur gradually throughout the MSOX series at 190 K, where full occupancy for NO is not achieved until ds18_190_. Further refinement to this work is required to establish the nature of these changes and the associated trigger. At RT, fully occupied NO is observed much earlier, at an accumulated dose of 0.18 MGy.

### 4.2. Insights into the T2Cu–NO complex in CuNiRs   

Our data provide the first room-temperature crystal structures of a CuNiR–NO complex generated *in situ* during catalysis, confirming that the side-on binding mode, which was observed in previous cryogenic structures of CuNiRs (Tocheva *et al.*, 2004 ▸; Antonyuk *et al.*, 2005 ▸; Horrell *et al.*, 2016 ▸), is not an artefact of sample cooling, cryoprotection or ligand soaking. Extensive theoretical work has previously examined the binding of NO to T2Cu in CuNiRs and overall has proposed that end-on binding should be energetically preferred over the side-on binding observed in the crystal structures. ENDOR/EPR experimental work on *Rhodobacter sphaeroides* CuNiR also suggested an end-on mode in solution (Usov *et al.*, 2006 ▸). Our data thus suggest either that CuNiR in room-temperature solution and CuNiR in the crystalline state produce different product geometries, which we consider to be unlikely, or that the theoretical studies have not yet fully represented the binding geometry *in vivo*. Our own DFT calculations have pointed towards a possible resolution of these differences between the predicted and experimental observations of NO binding by showing how dynamic re­organization from a top-hat nitrite-bound T2Cu(II) geometry to a side-on nitrite-bound T2Cu(I) geometry is required for optimal proton and electron delivery and bond cleavage.

The MSOX data also reveal, in both the 190 K and RT series, that the NO product can be released from the T2Cu site in crystals, allowing the rebinding of water to reform the resting state [T2Cu(II)(His)_3_·H_2_O]. In the 190 K series the crystal survives sufficiently long for the T2Cu-coordinated water to become dissociated, leaving a Cu(His)_3_ ligation that is consistent with a second reduction of the T2Cu site (Strange *et al.*, 1999 ▸; Howes *et al.*, 1994 ▸). Interestingly, there is no evidence in the electron-density maps for rebinding of nitrite to the [T2Cu(His)_3_·H_2_O] state following the release of NO, despite the high concentration of nitrite present in the crystal. It may be that nitrite rebinding is slowed or dynamically unfavourable at 190 K. Similarly, following the release of NO we do not observe a Cu(His_3_) species prior to the rebinding of water, implying that this step is fast.

### 4.3. Temperature-dependence of *in crystallo* enzyme catalysis and implications for MSOX experiments   

MSOX structural movies of *Ac*NiR catalysis have now been determined at 100 K (Horrell *et al.*, 2016 ▸), 190 K and RT. In the 100 K case, diffraction from the crystal was severely limited owing to radiation damage prior to full product release being observed and damage to the Asp_CAT_ residue was very apparent (lowered occupancy and negative *F*_obs_ − *F*_calc_ difference density). In contrast, and contrary to expectations, the 190 K series showed fewer overall signs of radiation damage through the majority of the MSOX series as measured from the electron density for the T2Cu-site region. This implies that the solvated electron-driven reactivity in the crystals is increased to a greater extent at 190 K than the general radiation damage that effects the loss of order and hence the loss of resolution. Work from several groups has indicated that a dynamical transition occurs in protein crystals around 180–220 K (summarized elegantly in Weik & Colletier, 2010 ▸), dependent on the properties of individual crystals. Below this temperature, anharmonic motions of the protein cease. Taking this together with our own observations, we propose that temperatures of around 190 K may represent a ‘sweet spot’ for X-ray-driven catalysis and the generation of MSOX structural movies. There is also evidence that solvent within the crystals occupies an ultraviscous state at higher temperatures (180–220 K window) with a liquid-like character, allowing long-range translational diffusion of water in channels (Weik & Colletier, 2010 ▸). A large number of structures (in this example 75) may be determined from atomic to high-medium resolution within the experimental dose limit^2^ (Warkentin *et al.*, 2014 ▸), allowing ‘fine slicing’ of the mechanism, while residue and ligand mobility is aided by increased dynamics within the crystal, allowing additional intermediates to be structurally determined.

At RT, the crystal lifetime is lowered by a factor of ∼100, largely owing to the ability of free radicals trapped at cryogenic temperatures to migrate rapidly. Consequently, far fewer structures may be measured within the dose limit and often to lower resolution, since the (electron-driven) catalysis may be outrun by the (electron- and free-radical-driven) general radiation damage, leading to a loss of resolution. However, there is an advantage in the much lower crystal viscosity (in the absence of cryoprotectants) and the greater freedom of the protein and ligands to move. Fewer intermediates were observed at RT compared with the MSOX series at 190 and 100 K, suggesting a powerful composite approach, in which MSOX data series at 190 K may be used to provide fine detail and to inform the interpretation of room-temperature MSOX data series, while both series provide ‘serial structural inputs’ to theoretical simulations.

Individual steps of the enzyme mechanism occur more rapidly than the data-collection time for each MSOX frame. As a consequence, each frame represents a ‘moving average’ of the structure, where highly populated states are represented in the electron-density map. A combination of new EIGER detector technology for rapid data collection (Casanas *et al.*, 2016 ▸) and more streamlined MSOX data collection with minimal dead time presents an exciting avenue for further development of this technique. This provides a parallel approach to XFEL-based SFX and serial femtosecond rotation crystallography (SF-ROX), where radiation damage-free structures are achieved but where electrons to drive the reaction could potentially be provided by incorporating NADH and activating using an Nd/YAG laser, as we have previously performed for solution experiments (Brenner *et al.*, 2009 ▸). 

## Supplementary Material

Supplementary Tables, Figures and Cartesian coordinates of the optimized structures reported in Figure 5.. DOI: 10.1107/S205225251800386X/lz5018sup1.pdf

PDB reference: copper nitrite reductase, 190 K data set 1, 5of5

PDB reference: 190 K data set 2, 5of6

PDB reference: 190 K data set 10, 5of7

PDB reference: 190 K data set 18, 5of8

PDB reference: 190 K data set 50, 5ofc

PDB reference: 190 K data set 69, 5ofd

PDB reference: 190 K data set 75, 5ofe

PDB reference: RT data set 1, 5off

PDB reference: RT data set 2, 5ofg

PDB reference: RT data set 3, 5ofh

PDB reference: RT data set 4, 5og2

PDB reference: RT data set 5, 5og3

PDB reference: RT data set 6, 5og4

PDB reference: RT data set 7, 5og5

PDB reference: RT data set 8, 5og6

PDB reference: RT data set 9, 5ogf

PDB reference: RT data set 10, 5ogg

## Footnotes

1 The room-temperature data series was measured using the standard 100 Hz readout of the PILATUS3 6M detector. An alternate mode of data collection using a limited detector area and readout at 200 Hz was also tested, resulting in the measurement of data sets in 1.5 s. However, the ∼14 s dead time between data sets negated the benefit of using this option.

2 The experimental dose limit at 180 K has been shown to be highly sample-dependent, with factors of 2 for the very stable thaumatin and of 8 for the much less stable 70S ribosome.
