# Supplementary material for: Enzyme catalysis captured using multiple structures from one crystal at varying temperatures
Source: IUCrJ. 2018 Mar 16;5(Pt 3):283–92. doi: 10.1107/S205225251800386X (PMC5929374; doi:10.1107/S205225251800386X)
Supplement: Supplementary file 1 [file m-05-00283-sup1.pdf]

IUCrJ

Volume 5 (2018)

Supporting information for article:

Enzyme catalysis captured using multiple structures from one crystal at varying temperatures

Sam Horrell, Demet Kekilli, Kakali Sen, Robin L. Owen, Florian S. N. Dworkowski, Svetlana V. Antonyuk, Thomas W. Keal, Chin W. Yong, Robert R. Eady, S. Samar Hasnain, Richard W. Strange and Michael A. Hough

Table S1 Data collection and crystallographic structure refinement statistics for the selected serial datasets from the nitrite-bound AcNiR structural movie at 190K. Data were processed using $CC_{1/2}$ >0.3 and I/σ(I) >1.0 (outer shell) cutoff.

**Structure**	$ds1_{190}$	$ds2_{190}$	$ds10_{190}$	$ds18_{190}$	$ds50_{190}$	$ds69_{190}$	$ds75_{190}$
Accumulated Dose (MGy)	0.24	0.48	2.40	4.89	21.65	46.51	59.05
Cell axis (Å)	94.9	94.9	94.9	95.0	95.0	95.0	95.0
Resolution (Å)	42.46-1.08 (1.10-1.08)	42.46-1.08 (1.10-1.08)	42.46-1.27 (1.29-1.27)	42.48-1.34 (1.36-1.34)	42.49-1.68 (1.71-1.68)	42.50-1.77 (1.81-1.77)	42.50-1.84 (1.88-1.84)
Unique reflections	120835	120809	75137	64147	32846	28154	25120
Redundancy	5.9 (3.1)	5.9 (3.2)	6.4 (6.1)	6.5 (6.0)	6.6 (6.3)	6.8 (6.9)	6.8 (7.0)
R_{pim} (%)	2.9 (62.3)	2.9 (72.9)	3.3 (69.7)	3.0 (74.6)	5.1 (79.0)	7.7 (78.0)	9.9 (79.2)
CC-1/2	0.99 (0.32)	0.99 (0.35)	0.99 (0.36)	0.99 (0.29)	0.99 (0.32)	0.99 (0.31)	0.99 (0.37)
I/σ(I)	13.7 (1.1)	13.5 (1.0)	12.2 (1.1)	13.1 (1.0)	10.6 (1.1)	8.3 (1.3)	7.7 (1.3)
Completeness (%)	99.4 (93.9)	99.4 (93.7)	100.0 (100.0)	99.9 (99.9)	99.9 (99.9)	100 (100)	100(100)
Wilson B-factor ($Å^2$)	8.0	8.6	12.1	14.5	29.1	29.7	29.3
**Refinement**							
R_{work}/R_{free} (%)	11.6/14.3	11.8/14.4	12.4/16.1	12.9/17.3	19.7/22.8	20.1/22.6	20.2/24.5
RMSD Bond Length (Å)	0.015	0.015	0.014	0.014	0.014	0.014	0.013
RMDS Bond Angle (°)	1.814	1.839	1.670	1.627	1.657	1.642	1.611
ML Based ESU (Å)	0.020	0.020	0.042	0.039	0.085	0.100	0.115
Av Protein B-factor ($Å^2$)	11.1	11.7	16.7	19.8	26.1	22.4	22.1
Av Water B-factor ($Å^2$)	25.1	25.8	32.4	34.1	31.2	25.6	25.0
No. waters	459	451	344	283	130	111	108
**Ramachandran (%)**							
Favoured Regions	99	99	99	99	99	99.7	99.7
Allowed Regions	1	1	1	1	1	0.3	1
PDB Code	5OF5	5OF6	5OF7	5OF8	5OFC	5OFD	5OFE

Table S2 Data collection and crystallographic structure refinement statistics for the selected serial datasets from the nitrite-bound AcNiR structural movie at room temperature. Data were processed using $CC_{1/2}$ ~ 0.5 I/ σ (I) >1.0 (outer shell) cut-off.

**Structure**	$ds1_{RT}$	$ds2_{RT}$	$ds3_{RT}$	$ds4_{RT}$	$ds5_{RT}$	$ds6_{RT}$	$ds7_{RT}$	$ds8_{RT}$	$ds9_{RT}$	$ds10_{RT}$
Accumulated Dose (MGy)	0.03	0.06	0.09	0.12	0.15	0.18	0.21	0.24	0.27	0.30
Cell axis (Å)	96.2	96.2	96.2	96.2	96.3	96.3	96.3	96.3	96.3	96.3
Resolution (Å) (outer shell)	48.11-1.41 (1.45-1.41)	55.56-1.49 (1.53-1.49)	48.12-1.58 (1.61-1.58)	55.57-1.64 (1.68-1.64)	48.13-1.70 (1.74-1.70)	55.58-1.76 (1.81-1.76)	48.13-1.82 (1.87-1.82)	48.13-1.85 (1.90-1.85)	48.13-1.88 (1.93-1.88)	48.12-1.91 (1.96-1.91)
Unique reflections	57140	48510	40744	36457	32772	29589	26780	25531	24354	23248
Redundancy	3.3 (3.3)	3.3 (3.3)	3.3 (3.3)	3.3 (3.3)	3.3 (3.3)	3.3 (3.2)	3.3 (3.2)	3.3 (3.2)	3.3 (3.1)	3.3 (3.2)
R_{pim} (%)	7.9 (57.0)	7.7 (57.8)	7.9 (53.3)	8.1 (55.2)	8.6 (54.9)	8.9 (51.3)	9.4 (48.7)	9.8 (46.8)	10.4 (46.0)	11.0 (48.7)
CC-1/2	0.99 (0.50)	0.99 (0.50)	0.99 (0.54)	0.99 (0.53)	0.99 (0.54)	0.99 (0.51)	0.99 (0.55)	0.99 (0.55)	0.99 (0.60)	0.99 (0.58)
I/σ(I)	5.1 (1.2)	5.3 (1.2)	5.2 (1.3)	5.2 (1.3)	5.0 (1.3)	4.9 (1.3)	4.9 (1.5)	4.7 (1.4)	4.5 (1.4)	4.4 (1.4)
Completeness (%)	99.7 (99.8)	99.7 (99.8)	99.6 (99.7)	99.6 (99.8)	99.6 (99.6)	99.6 (99.7)	99.5 (99.7)	99.5 (99.3)	99.5 (97.6)	99.6 (97.5)
Wilson B-factor ($Å^2$)	10.9	13.6	17.5	16.3	17.6	20.0	21.8	23.0	22.8	24.7
**Refinement**										
R_{work}/R_{free} (%)	16.0/18.4	15.9/18.4	15.4/17.8	16.4/19.0	16.6/19.4	17.2/20.3	17.4/20.9	17.7/22.3	18.3/21.6	18.6/23.0
RMSD Bond Length (Å)	0.014	0.014	0.014	0.014	0.014	0.014	0.014	0.013	0.013	0.013
RMDS Bond Angle (°)	1.68	1.67	1.67	1.65	1.68	1.69	1.72	1.65	1.63	1.68
ML Based ESU (Å)	0.055	0.064	0.072	0.083	0.093	0.105	0.118	0.128	0.133	0.144
Av Protein B-factor ($Å^2$)	16.8	19.1	21.5	21.1	21.4	21.9	22.2	22.1	22.0	22.1
Av Water B-factor ($Å^2$)	27.7	29.6	31.7	30.8	29.9	29.5	28.7	28.0	27.2	26.4
No. waters	186	184	159	161	155	150	138	131	124	122
**Ramachandran (%)**										
Favoured Regions	99	99	98.8	98.8	99	99	98.8	98.5	98.2	99
Allowed Regions	1	1	1.2	1.2	1	1	1.2	1.5	1.8	1
PDB Code	5OFF	5OFG	50FH	5OG2	5OG3	5OG4	5OG5	5OG6	5OGF	5OGG

Table S3 Cu-ligand distances and angles at the T1Cu site for selected frames of the 190K series movie.

**Structure**	**$ds1_{190}$**	**$ds2_{190}$**	**$ds10_{190}$**	**$ds18_{190}$**	**$ds50_{190}$**	**$ds69_{190}$**	**$ds75_{190}$**
**Resolution (Å)**	1.08	1.08	1.27	1.34	1.68	1.77	1.84
**Bond length (Å)**							
T1Cu-His145Nδ1	2.02	2.05	2.09	2.13	2.11	2.10	2.07
T1Cu-His95Nδ1	2.01	2.02	2.04	2.06	2.08	2.13	2.14
T1Cu-Cys136 Sγ	2.19	2.20	2.17	2.15	2.18	2.23	2.19
T1Cu-Met150 Sδ	2.54	2.55	2.59	2.61	2.66	2.58	2.60
**Bond angle (°)**							
His95Nδ1-T1Cu- His145Nδ1	101.3	101.8	101.7	103.3	102.0	104.1	102.7
His145Nδ1-T1Cu- Cys136 Sγ	107.7	108.6	109.4	107.5	109.1	107.8	109.9
Cys136 Sγ-T1Cu- Met150 Sδ	107.1	107.0	107.4	108.3	108.2	109.8	109.6
Met150 Sδ-T1Cu- His95Nδ1	84.9	85.3	84.8	84.8	84.5	84.6	84.7
Cys136 Sγ-T1Cu- His95Nδ1	131.9	129.9	129.7	130.1	128.9	128.2	128.0
Met150 Sδ-T1Cu- His145Nδ1	124.5	125.1	124.1	121.4	124.4	122.6	121.9

Table S4 Cu-ligand distances and angles at the T1Cu site for selected frames of the RT movie

**Structure**	$ds1_{RT}$	$ds2_{RT}$	$ds4_{RT}$	$ds6_{RT}$	$ds8_{RT}$	$ds10_{RT}$
**Resolution (Å)**	1.41	1.49	1.64	1.76	1.85	1.91
**Bond length (Å)**						
T1Cu-His145Nδ1	2.02	2.04	2.03	2.04	2.02	2.03
T1Cu-His95Nδ1	2.04	2.09	2.06	2.07	2.05	2.05
T1Cu-Cys136 Sγ	2.20	2.20	2.23	2.26	2.23	2.24
T1Cu-Met150 Sδ	2.51	2.50	2.47	2.42	2.41	2.44
**Bond angle (°)**						
His95Nδ1-T1Cu- His145Nδ1	99.2	99.9	100.5	100.6	97.7	100.7
His145Nδ1-T1Cu- Cys136 Sγ	105.3	106.5	104.2	103.5	103.5	104.4
Cys136 Sγ-T1Cu- Met150 Sδ	106.2	107.1	107.2	109.3	107.3	107.4
Met150 Sγ-T1Cu- His95Nδ1	87.2	86.7	86.1	86.4	89.0	86.0
Cys136 Sγ-T1Cu- His95Nδ1	135.1	132.5	133.9	132.5	131.5	134.1
Met150 Sδ-T1Cu- His145Nδ1	126.8	126.3	128.2	126.3	127.3	127.1

Table S5 T2Cu-ligand distances for selected serial datasets at 190 K for nitrite soaked *Ac*NiR. In $ds1_{190}$ the Asp98 residue is partially (~35%) in the gatekeeper position.

Structure	$ds1_{190}$	$ds2_{190}$	$ds10_{190}$	$ds18_{190}$	$ds50_{190}$	$ds69_{190}$	$ds75_{190}$
**Resolution (Å)**	1.08	1.08	1.27	1.34	1.68	1.77	1.84
**Bond lengths (Å)**							
His100Nε2	2.01	2.02	2.05	2.04	2.06	2.07	2.11
His135Nε2	2.06	2.05	2.06	2.07	2.06	2.07	2.05
His306Nε2	2.00	2.01	2.05	2.05	2.09	2.09	2.12
His255Nε2	4.18	4.19	4.20	4.12	4.15	4.12	4.21
NO_2 (O1/O2)	2.03/1.97	2.15/1.98	2.22/2.02	-	-	-	-
O/N (NO)	-	-	2.20/1.98	2.20/1.90	-	-	-
H_2O	-	-	-	-	1.93	-	-
Asp98Oδ2-NO_2	2.26	2.19	2.27	-	-	-	-
Asp98-NO	-	-	2.51	2.69	-	-	-
Asp98-H_2O	-	-	-	-	3.08	-	

Table S6 T2Cu-ligand distances (Å) for selected serial datasets at RT for nitrite soaked *Ac*NiR.

Structure	ds1	ds2	ds4	ds6	ds8	ds10
**Resolution (Å)**	1.41	1.49	1.64	1.76	1.85	1.91
**Bond lengths (Å)**						
His100Nε2	2.04	2.04	2.07	2.06	2.05	2.06
His135Nε2	2.08	2.07	2.06	2.06	2.07	2.06
His306Nε2	2.01	2.03	2.08	2.04	2.07	2.03
His255Nε2	4.09	4.07	4.05	4.02	4.02	4.05
NO_2a (O1/O2)	1.97/1.91	1.96/1.91	2.16/2.03	-	-	-
O/N (NO)	-	-		2.35/2.08	2.49/2.00	-
H_2O	-	-	-	-	-	2.04
Asp98Oδ2-NO_2	2.20	2.23	2.22	-	-	-
Asp98Oδ2-NO	-	-	-	2.81	2.63	-

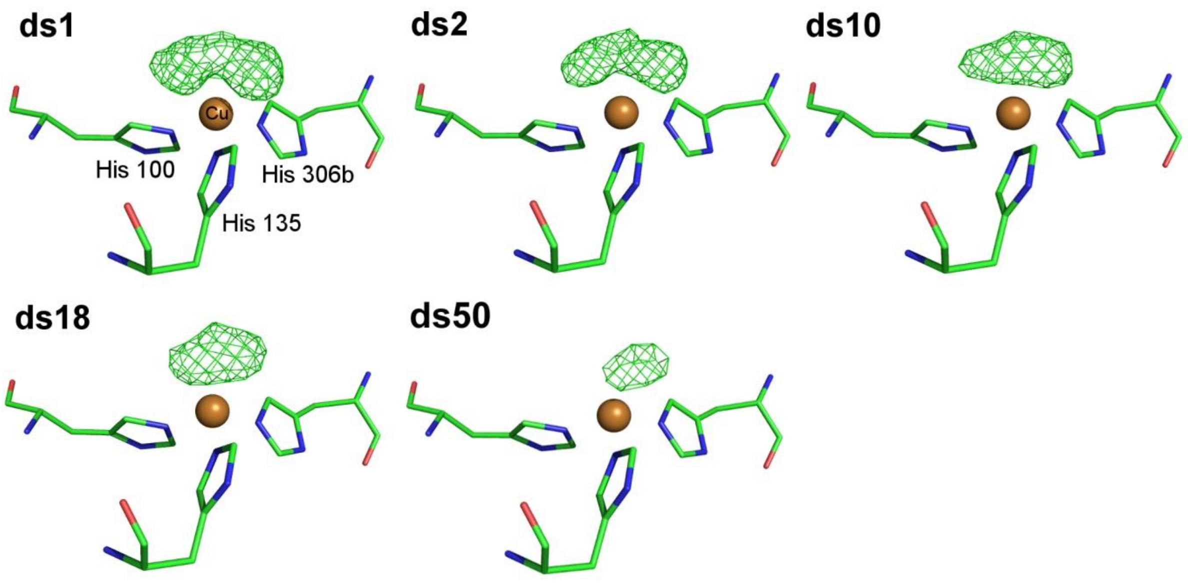

Figure S1 Ligand binding at the T2Cu site for 190 K series. 3σ, weighted electron density Fo-Fc difference maps are shown for the datasets reported in the manuscript and Table S1.

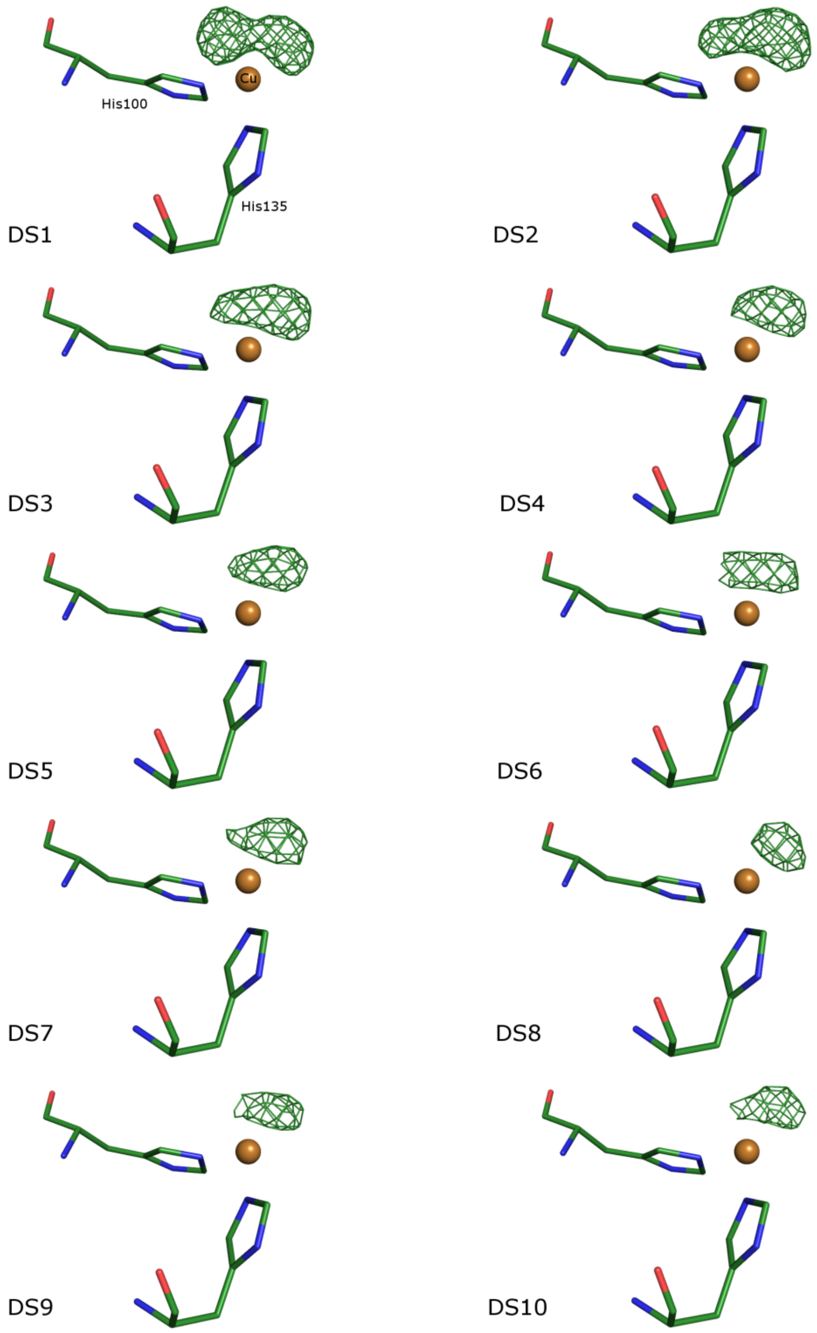

Figure S2 Ligand binding at the T2Cu site for RT series. 3σ, weighted electron density Fo-Fc difference maps are shown for the datasets reported in the manuscript and Table S2.

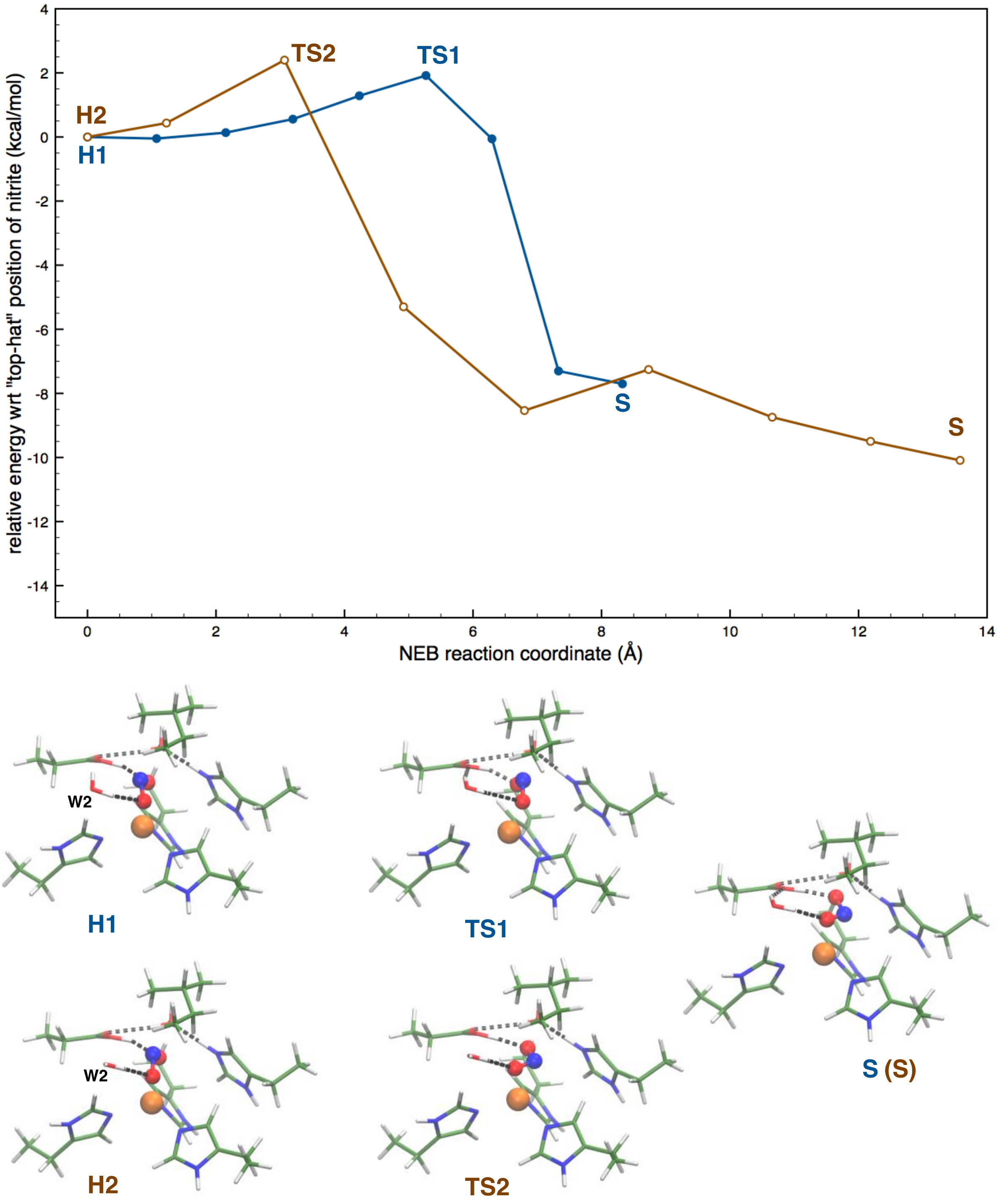

Figure S3 Optimised minimum energy paths between the top-hat and side-on orientation of nitrite at the T2Cu(I) site. Initial top-hat geometries are labelled H1 and H2, with corresponding paths in blue and brown respectively via transition states TS1 and TS2. The structure of the final side-on geometry labelled S is the same for both pathways, while the position of S along the x-axis varies due to the differences in reaction coordinate path lengths. The minimum energy path (MEP) was generated using the geometries in Fig. 5, giving a barrier of 1.9 - 2.4 kcal/mol, depending on the

position of water molecule W2. The barrier is estimated from the initial top-hat orientation of nitrite and the highest point on the path. The barrier to transition is low and hence reorientation of nitrite is likely to take place when the Cu is reduced. Although the barriers calculated from the different top-hat starting geometries are similar, the MEPs are somewhat different. The approximate transition state TS1 in the first path is an early TS and resembles a top-hat orientation, whereas TS2 in the second path is a late TS and is closer to the side-on orientation. The different positioning of W2 influences the nature of the transition state and reorientation of W2 causes the small distortion in the energy pathway observed at a reaction coordinate of approximately 7 Å in TS2. Despite these differences, the barrier is low enough in both cases to suggest that the transition from top-hat to side-on nitrite is straightforward in the Cu(I) state.

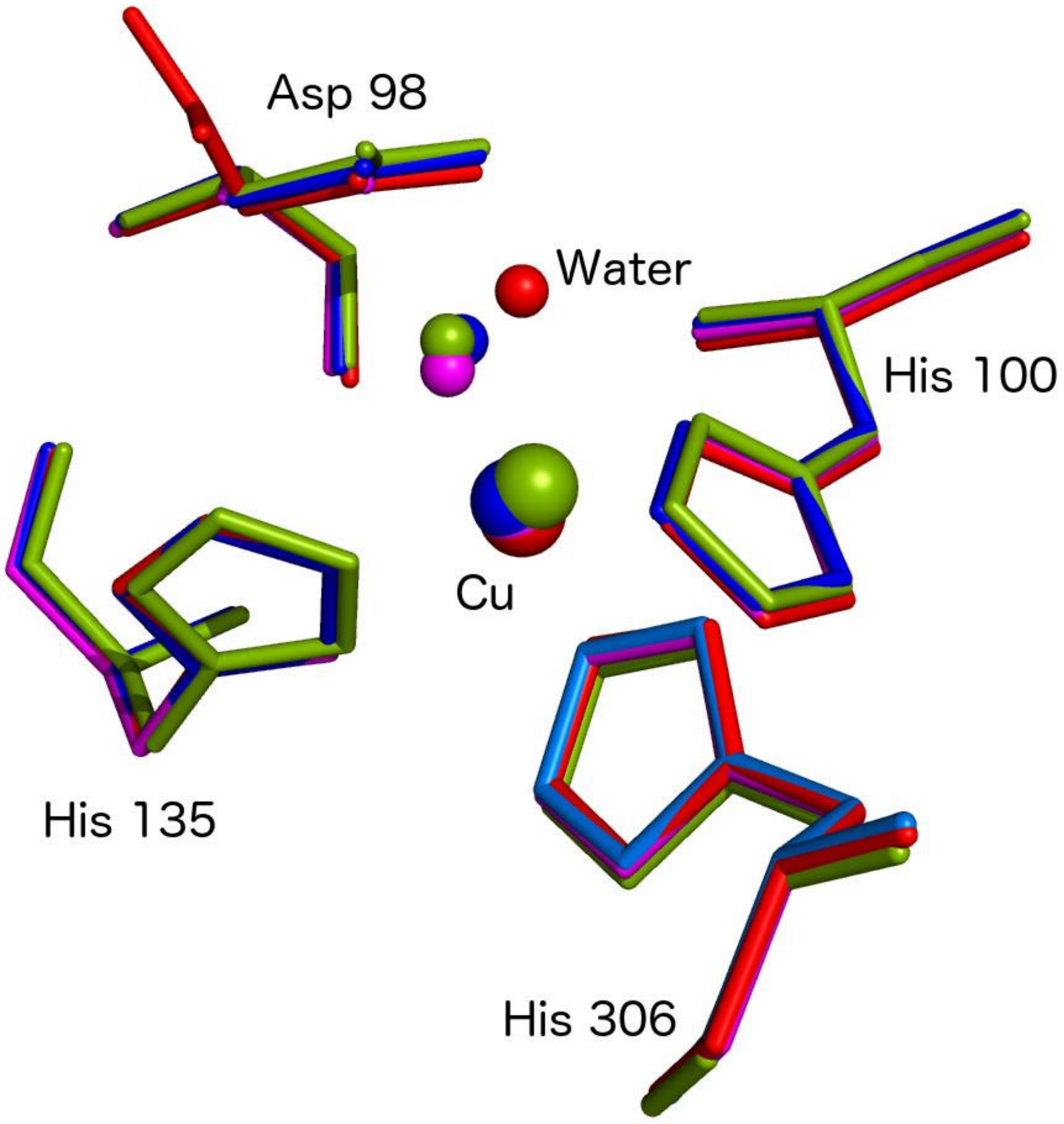

Figure S4 Superposition of the T2Cu-water structures from ds50$_{190}$, ds10RT with previously determined MSOX series at 100K [magenta, PDB 5i6p, (Horrell *et al*., 2017)] and the structure of the resting state enzyme [red, PDB 2bw4 [(Antontyuk *et al*., 2005)], which was also determined at 100K. Note that a longer T2Cu-water separation is observed in the resting state enzyme together with a dual conformation of Asp98, not observed in our MSOX structures.

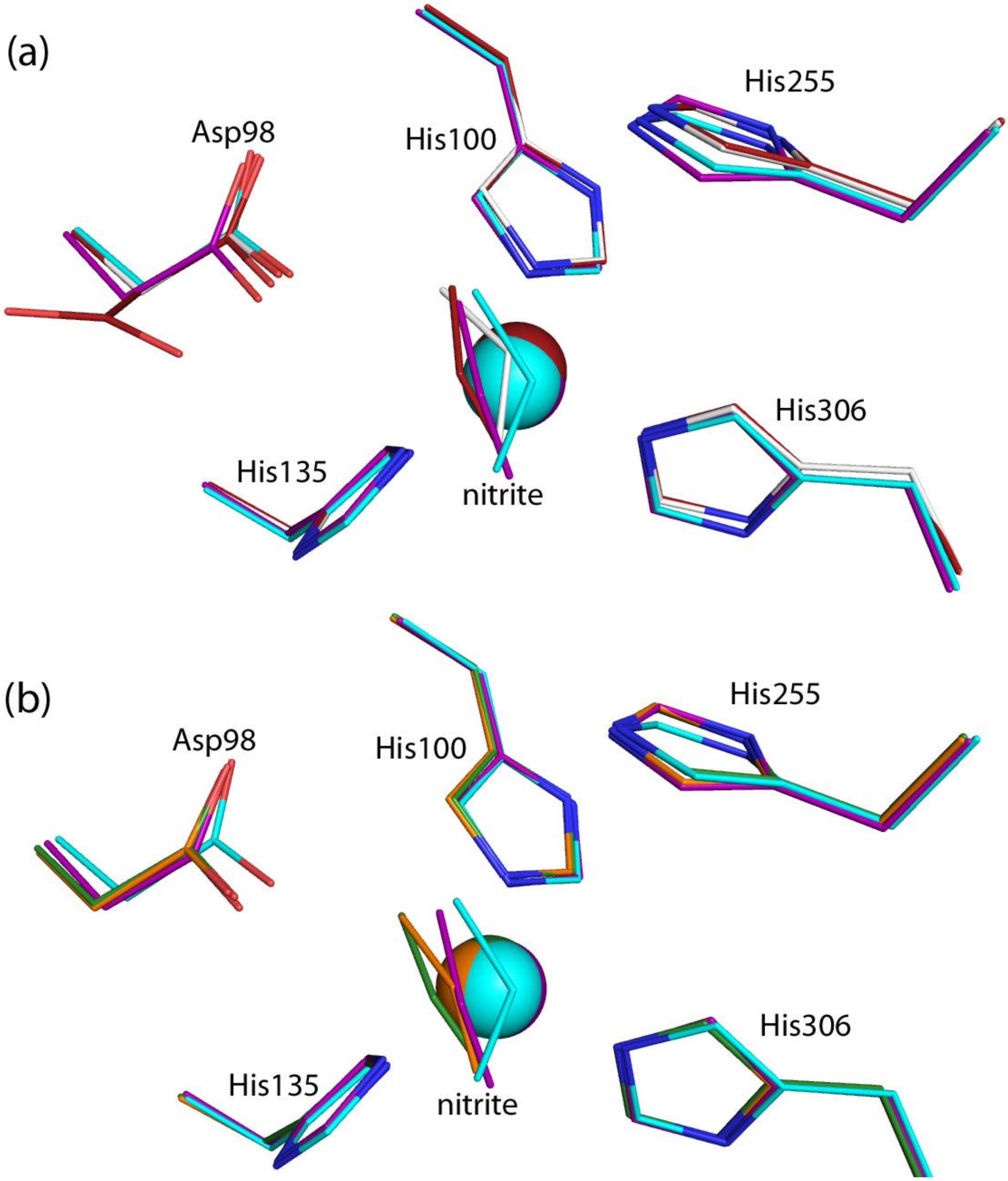

Figure S5 Superposition of T2Cu site structures for nitrite bound *Ac*NiR. The MSOX serial structures at 190K (a) and RT (b) are shown for ‘top-hat’ $ds1_{190}$ (red)/ $ds1_{RT}$ (green) and ‘side-on’ $ds3_{190}$ (white)/ $ds3_{RT}$ (orange) and are compared in both panels to the *Af*NiR nitrite bound structures obtained using SFX (purple) and SR methods (cyan) (Fukuda *et al.*, 2016).

References

Horrell, S., Antonyuk, S. V., Eady, R. R., Hasnain, S. S., Hough, M. A. & Strange, R. W. (2016). *IUCrJ* **3**, 271-281.

Antonyuk, S. V., Strange, R. W., Sawers, G., Eady, R. R. & Hasnain, S. S. (2005). *Proc. Natl. Acad. Sci. USA* **102**, 12041-12046.

Fukuda, Y., Tse, K. M., Nakane, T., Nakatsu, T., Suzuki, M., Sugahara, M., Inoue, S., Masuda, T., Yumoto, F., Matsugaki, N., Nango, E., Tono, K., Joti, Y., Kameshima, T., Song, C., Hatsui, T., Yabashi, M., Nureki, O., Murphy, M. E. P., Inoue, T., Iwata, S. & Mizohata, E. (2016). *Proc. Natl. Acad. Sci. USA* **113**, 2928-2933.

Cartesian coordinates of the optimized structures reported in Fig5

Fig 5A. Optimized top-hat NO_2^- binding to T2Cu(II)

C -82.440002 -40.873001 27.580000
H -82.534568 -40.833787 26.485481
H -81.380789 -40.746125 27.847591
H -82.762839 -41.865092 27.929701
C -83.307999 -39.764999 28.209999
H -84.366831 -39.875933 27.910432
H -83.009083 -38.763289 27.857030
C -83.325996 -39.710999 29.767000
O -82.828003 -40.604000 30.461000
O -83.901001 -38.724998 30.306999
H -83.949195 -38.792677 31.281376
C -83.183998 -43.875999 33.056000
H -82.500061 -44.660753 33.408294
H -83.141060 -43.868924 31.955544
H -82.793325 -42.907304 33.408082
C -84.578003 -44.123001 33.558998
H -84.580209 -44.216212 34.661323
H -84.946751 -45.096334 33.184631
C -85.573997 -43.063999 33.173000
N -86.836998 -43.075001 33.702000
H -87.210143 -43.833453 34.266520
C -87.503998 -42.029999 33.297001
H -88.533416 -41.799778 33.560534
N -86.737000 -41.311001 32.509998
C -85.509003 -41.931000 32.424000
H -84.690045 -41.543080 31.821110
C -87.307999 -42.450001 25.966999
H -87.736139 -43.133447 25.219478
H -86.733395 -41.676808 25.432876
H -86.609669 -43.018598 26.600033
C -88.452003 -41.827000 26.822001
H -89.017312 -42.639409 27.304658
H -89.169527 -41.323476 26.150961
C -88.031998 -40.875000 27.884001
N -87.778000 -39.547001 27.646999
H -87.867912 -39.082022 26.749194
C -87.445999 -38.958000 28.794001
H -87.206444 -37.898579 28.908071
N -87.435997 -39.849998 29.747999
C -87.806999 -41.056999 29.201000
H -87.886000 -41.965860 29.791713
C -87.014998 -37.764001 38.837003
H -87.777313 -37.261613 39.450010
H -86.634881 -38.630796 39.399242
H -86.180678 -37.063120 38.686283
C -87.622001 -38.194001 37.476003
H -88.511019 -38.824145 37.643228
H -87.973387 -37.298497 36.939053
C -86.665999 -38.933000 36.615003
N -86.716002 -40.285001 36.488000
H -87.374372 -40.888313 36.971564
C -85.716002 -40.702001 35.750001
H -85.490532 -41.734772 35.499421
N -85.032996 -39.669000 35.336000
H -84.266561 -39.723655 34.622278
C -85.624999 -38.539002 35.853002
H -85.246406 -37.545164 35.637236
C -82.637999 -36.596002 34.766999
H -81.538914 -36.629375 34.704321
H -82.898809 -36.288952 35.794657
H -83.012472 -37.618572 34.596787
C -83.222999 -35.639001 33.717000
H -82.719188 -34.661670 33.840523
C -84.718001 -35.410001 33.900003
H -84.866386 -34.937790 34.889504
H -85.210090 -36.391565 33.958573
C -85.439002 -34.570001 32.849999
H -86.486614 -34.404255 33.147131
H -84.963044 -33.578785 32.749948
H -85.455685 -35.036792 31.856344
C -82.869002 -36.174001 32.349000
H -81.777588 -36.229911 32.210080
H -83.275749 -37.188568 32.221122
H -83.268429 -35.557428 31.530148
C -92.029998 -37.131001 35.453000
H -92.493683 -36.835159 36.405765
H -91.448153 -36.276785 35.073520
H -92.849363 -37.321631 34.741327
C -91.142997 -38.359001 35.634999
H -91.744522 -39.211156 35.998990
H -90.389872 -38.164614 36.412545
C -90.410003 -38.770000 34.396001
N -91.042999 -39.223999 33.273000
H -92.049537 -39.316741 33.168009
C -90.138000 -39.576000 32.362001
H -90.369414 -39.970501 31.373727
N -88.948997 -39.303001 32.834001
C -89.089996 -38.806000 34.104001
H -88.235521 -38.487526 34.689357
Cu -87.226997 -39.501999 31.761000
O -87.071031 -37.513878 31.518011
N -85.920404 -37.374236 31.991404
O -85.465330 -38.443839 32.475076
O -82.957636 -40.118408 33.394141
H -82.053469 -39.897154 33.662814
H -82.879933 -40.346796 32.437524
O -86.630413 -35.989921 29.152343
H -87.032050 -35.714498 29.988372
H -86.274180 -35.179215 28.765219

Figure 5B. Optimized top-hat NO_2^- binding to T2Cu(I)

C -82.440002 -40.873001 27.580000
H -82.553613 -40.854541 26.486182
H -81.376944 -40.734316 27.828036
H -82.747941 -41.861454 27.953165
C -83.307999 -39.764999 28.209999
H -84.368700 -39.882426 27.921739
H -83.017603 -38.762827 27.851337
C -83.325996 -39.710999 29.767000
O -82.828003 -40.604000 30.461000
O -83.901001 -38.724998 30.306999
H -84.276054 -38.783804 31.242805
C -83.183998 -43.875999 33.056000
H -82.498391 -44.667788 33.391032
H -83.146372 -43.843801 31.955569
H -82.790131 -42.912667 33.419941
C -84.578003 -44.123001 33.558998
H -84.572320 -44.222245 34.661810
H -84.939422 -45.100442 33.185084
C -85.573997 -43.063999 33.173000
N -86.836998 -43.075001 33.702000
H -87.204275 -43.837134 34.263634
C -87.503998 -42.029999 33.297001
H -88.534018 -41.796450 33.559755
N -86.737000 -41.311001 32.509998
C -85.509003 -41.931000 32.424000
H -84.695035 -41.543674 31.815725
C -87.307999 -42.450001 25.966999
H -87.727599 -43.139313 25.218624
H -86.734182 -41.674508 25.434537
H -86.606435 -43.010178 26.604421
C -88.452003 -41.827000 26.822001
H -89.014132 -42.642711 27.304021
H -89.171656 -41.329909 26.146916
C -88.031998 -40.875000 27.884001
N -87.778000 -39.547001 27.646999
H -87.871001 -39.087985 26.747460
C -87.445999 -38.958000 28.794001
H -87.193452 -37.903377 28.901189
N -87.435997 -39.849998 29.747999
C -87.806999 -41.056999 29.201000
H -87.885216 -41.960563 29.801509
C -87.014998 -37.764001 38.837003
H -87.771968 -37.254166 39.451377
H -86.638020 -38.631601 39.400964
H -86.175252 -37.069813 38.683872
C -87.622001 -38.194001 37.476003
H -88.511787 -38.823486 37.641024
H -87.972775 -37.299596 36.937529
C -86.665999 -38.933000 36.615003
N -86.716002 -40.285001 36.488000
H -87.394661 -40.888208 36.940017
C -85.716002 -40.702001 35.750001
H -85.514843 -41.731007 35.463275
N -85.032996 -39.669000 35.336000
H -84.278236 -39.723883 34.599361
C -85.624999 -38.539002 35.853002
H -85.256993 -37.545413 35.620241
C -82.637999 -36.596002 34.766999
H -81.537781 -36.623962 34.710746
H -82.908635 -36.297121 35.795032
H -83.013151 -37.617154 34.591591
C -83.222999 -35.639001 33.717000
H -82.714424 -34.663013 33.843840
C -84.718001 -35.410001 33.900003
H -84.870432 -34.947712 34.894868
H -85.208717 -36.392993 33.932781
C -85.439002 -34.570001 32.849999
H -86.496185 -34.440994 33.128748
H -84.986439 -33.564414 32.771946
H -85.424994 -35.020561 31.849881
C -82.869002 -36.174001 32.349000
H -81.777592 -36.272143 32.225750
H -83.320446 -37.164730 32.198005
H -83.232002 -35.530626 31.533784
C -92.029998 -37.131001 35.453000
H -92.512526 -36.840563 36.399045
H -91.442713 -36.275007 35.085403
H -92.834192 -37.318898 34.722963
C -91.142997 -38.359001 35.634999
H -91.748972 -39.205570 36.007648
H -90.389857 -38.162864 36.412712
C -90.410003 -38.770000 34.396001
N -91.042999 -39.223999 33.273000
H -92.048384 -39.319852 33.174504
C -90.138000 -39.576000 32.362001
H -90.364381 -39.971720 31.373151
N -88.948997 -39.303001 32.834001
C -89.089996 -38.806000 34.104001
H -88.230501 -38.483099 34.680597
Cu -87.226997 -39.501999 31.761000
O -87.004383 -37.044176 31.685101
N -85.794727 -37.244349 31.740839
O -85.467335 -38.388612 32.226122
O -83.021031 -40.069877 33.439637
H -82.167434 -39.640285 33.593687
H -83.015653 -40.268520 32.472832
O -86.551797 -35.876980 29.175378
H -86.974811 -35.972348 30.047739
H -85.639361 -36.129279 29.380619

Figure 5C. Optimized side-on NO_2^- binding to T2Cu(I)

C -82.504997 -40.918999 27.530001

H	-82.505981	-40.910737	26.429305
H	-81.469552	-40.821580	27.889059
H	-82.887189	-41.890758	27.879274
C	-83.389999	-39.763000	28.087999
H	-83.035959	-38.779169	27.736842
H	-84.429191	-39.850564	27.722854
C	-83.487999	-39.667000	29.632000
O	-82.941002	-40.499001	30.406000
O	-84.134003	-38.668999	30.097000
H	-84.342349	-38.740121	31.104393
C	-83.234001	-43.950001	33.138000
H	-82.556390	-44.731706	33.509995
H	-83.192532	-43.962307	32.037706
H	-82.844264	-42.972287	33.465812
C	-84.642998	-44.176998	33.646000
H	-84.635143	-44.267932	34.749564
H	-85.012050	-45.155227	33.281322
C	-85.627998	-43.118000	33.257999
N	-86.897003	-43.122002	33.780998
H	-87.280964	-43.874207	34.343260
C	-87.550003	-42.067001	33.328999
H	-88.578080	-41.809390	33.576278
N	-86.748001	-41.366001	32.562000
C	-85.545998	-42.019001	32.474998
H	-84.719935	-41.634230	31.875497
C	-87.425003	-42.456001	25.958000
H	-87.817375	-43.157317	25.205814
H	-86.848754	-41.678513	25.431455
H	-86.729240	-43.000892	26.614326
C	-88.589996	-41.841999	26.781000
H	-89.150601	-42.651633	27.273658
H	-89.303856	-41.351063	26.096088
C	-88.169998	-40.867001	27.841999
N	-87.926003	-39.536999	27.610001
H	-87.947354	-39.097272	26.696558
C	-87.612999	-38.924999	28.750000
H	-87.309375	-37.880181	28.858785
N	-87.654999	-39.806000	29.724001
C	-88.017998	-41.027000	29.186001
H	-88.120063	-41.922078	29.794484
C	-87.108003	-37.827001	38.902000
H	-87.867061	-37.342147	39.533650
H	-86.707948	-38.694691	39.450288
H	-86.282549	-37.114304	38.754986
C	-87.721002	-38.241002	37.553001
H	-88.104341	-37.345222	37.039296
H	-88.596463	-38.890585	37.721770
C	-86.781999	-38.941002	36.636002
N	-86.745004	-40.299000	36.528000
H	-87.341792	-40.941849	37.037607
C	-85.771004	-40.654999	35.721001
H	-85.499936	-41.669933	35.439772
N	-85.202004	-39.581001	35.259998
H	-84.494757	-39.578699	34.485636
C	-85.849000	-38.490002	35.793999
H	-85.553442	-37.478446	35.534332
C	-82.759004	-36.749001	34.784999
H	-81.657714	-36.719534	34.776069
H	-83.092572	-36.461613	35.797569
H	-83.067121	-37.786789	34.589838
C	-83.339997	-35.807999	33.698001
H	-82.894844	-34.807418	33.864609
C	-84.857003	-35.660999	33.801997
H	-85.310956	-36.661596	33.722024
H	-85.101062	-35.277756	34.813257
C	-85.558999	-34.777000	32.762000
H	-85.473678	-35.192676	31.750501
H	-85.133284	-33.758450	32.755012
H	-86.631546	-34.690757	32.998702
C	-82.927003	-36.301998	32.339999
H	-81.828986	-36.324167	32.236682
H	-83.308830	-37.315485	32.141649
H	-83.306507	-35.665506	31.526852
C	-92.123002	-37.183999	35.490002
H	-92.563905	-36.909301	36.460560
H	-91.578076	-36.311831	35.096216
H	-92.957144	-37.394611	34.800956
C	-91.193002	-38.391000	35.625000
H	-91.757399	-39.254616	36.022232
H	-90.409310	-38.173424	36.365290
C	-90.497003	-38.782002	34.352001
N	-91.129998	-39.284001	33.244999
H	-92.133522	-39.399224	33.149743
C	-90.226998	-39.557000	32.308998
H	-90.449971	-39.957936	31.321325
N	-89.038003	-39.257000	32.768002
C	-89.182000	-38.770001	34.040001
H	-88.329555	-38.427659	34.615756
Cu	-87.295998	-39.577999	31.743999
O	-86.395604	-37.631934	31.756515
N	-85.974322	-38.316937	32.729109
O	-84.818489	-38.848798	32.563189
O	-82.909525	-40.425341	33.650048
H	-81.964438	-40.357641	33.841974
H	-82.970913	-40.294432	32.682010
O	-85.940854	-36.397618	29.208814
H	-85.241771	-37.064813	29.326426
H	-86.289517	-36.351615	30.112342
